# Ocular surface squamous neoplasia masquerading as pseudoepitheliomatous hyperplasia in chronic vernal keratoconjunctivitis

**DOI:** 10.3205/oc000234

**Published:** 2024-01-24

**Authors:** Aditi Ghosh Dastidar, Deepika Khedia, Sugandha Goel

**Affiliations:** 1 Cornea and Refractive Services, Trenetralaya, Kolkata, India; 2 Iris Superspeciality Eye Hospital, Ranchi, India; 3 JP Eye Hospital, a unit of Dr Agarwals Eye Hospital, Mohali, India

**Keywords:** OSSN, pseudoepitheliomatous hyperplasia, vernal keratoconjunctivitis

## Abstract

We report a rare case of ocular surface squamous neoplasia (OSSN) masquerading as pseudoepitheliomatous hyperplasia in chronic vernal keratoconjunctivitis (VKC). A 24-year-old man presented with a history of bilateral VKC since childhood with a superior limbal mass in the right eye. There was a history of use of intermittent corticosteroids in the past. He underwent impression cytology followed by excision biopsy with wide margins (no touch technique), cryotherapy and amniotic membrane transplantation. Histopathological analysis confirmed the diagnosis of OSSN with mild to moderate dysplasia. This case highlights the importance of strong clinical suspicion and detailed cytological and histopathological examination for early detection and management of OSSN.

## Introduction

Ocular surface squamous neoplasia (OSSN) describes a spectrum of conjunctival and corneal epithelial neoplasia manifesting as dysplasia, carcinoma-in-situ and squamous cell carcinoma [1]. The two main risk factors are UV-B light and human papilloma virus [2]. OSSN is a slow growing tumour of low-grade malignancy which rarely metastases. Three morphological types are described as gelatinous, leukoplakic and papilliform type, the gelatinous type being the most common. OSSN has been reported to present as corneal ulcer, chronic blepharoconjunctivitis, pterygium, necrotizing scleritis, superior limbic keratoconjunctivitis and pseudoepitheliomatous hyperplasia (PEH) [3], [4], [5], [6], [7], [8]. We herein report a rare case of OSSN mimicking PEH in chronic Vernal keratoconjunctivitis (VKC). 

## Case description

A 24-year-old man was referred to the department of cornea with a history of bilateral VKC since childhood with intermittent use of topical steroids in the past. Previous medical and ocular surgical history was unremarkable.

**Figure 1:**
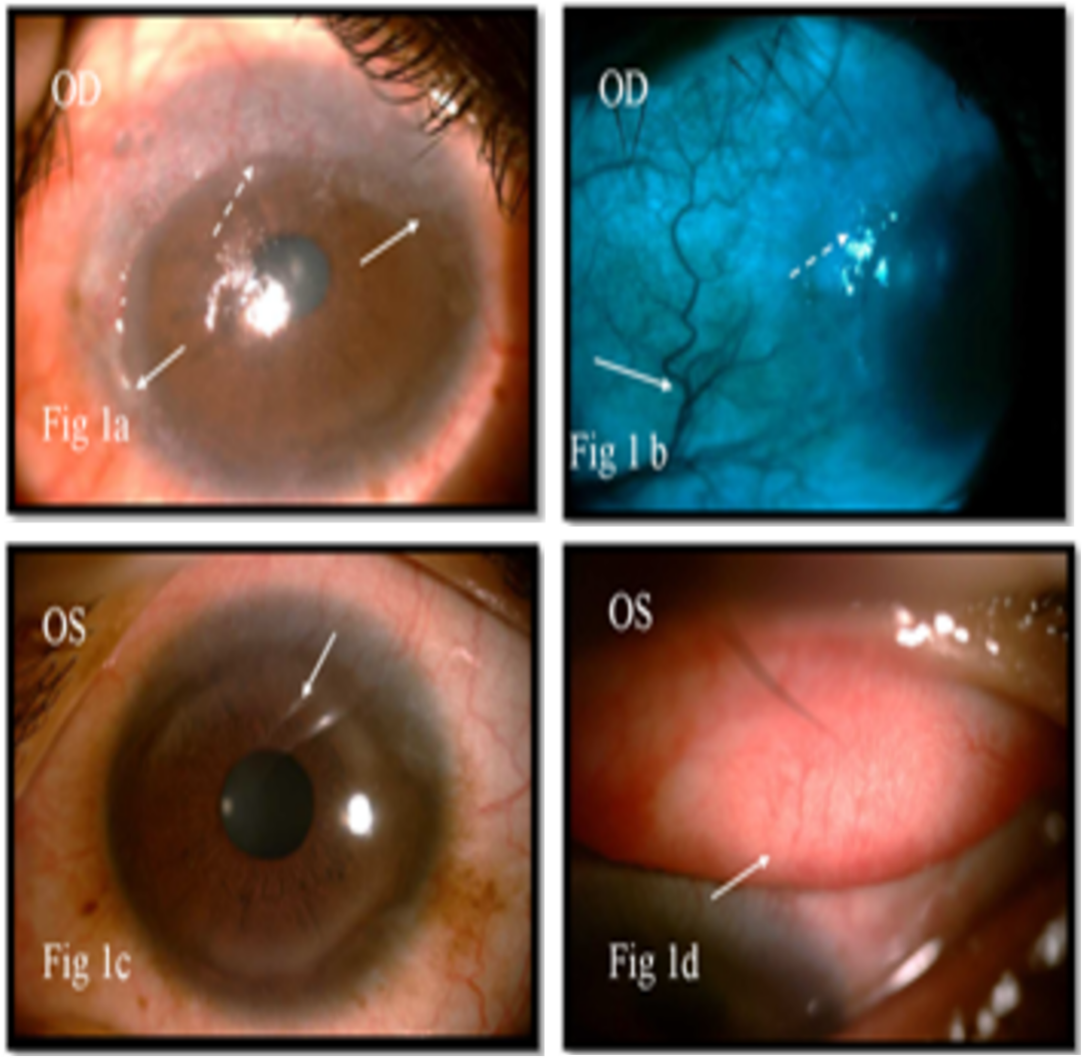
Right eye showing gelatinous lesion extending from 7 o’clock to 2 o’clock (arrows) with 3 mm corneal encroachment (interrupted arrow) with vascular fronds on surface, with feeder vessels (arrow) (a). Rose Bengal stain positivity (interrupted arrow) of surface in green filter with feeder vessel (arrow) (b). Left eye showing no conjunctival congestion, pseudogerontoxon with no papillary and limbal activity (arrows) (c, d).

**Figure 2:**
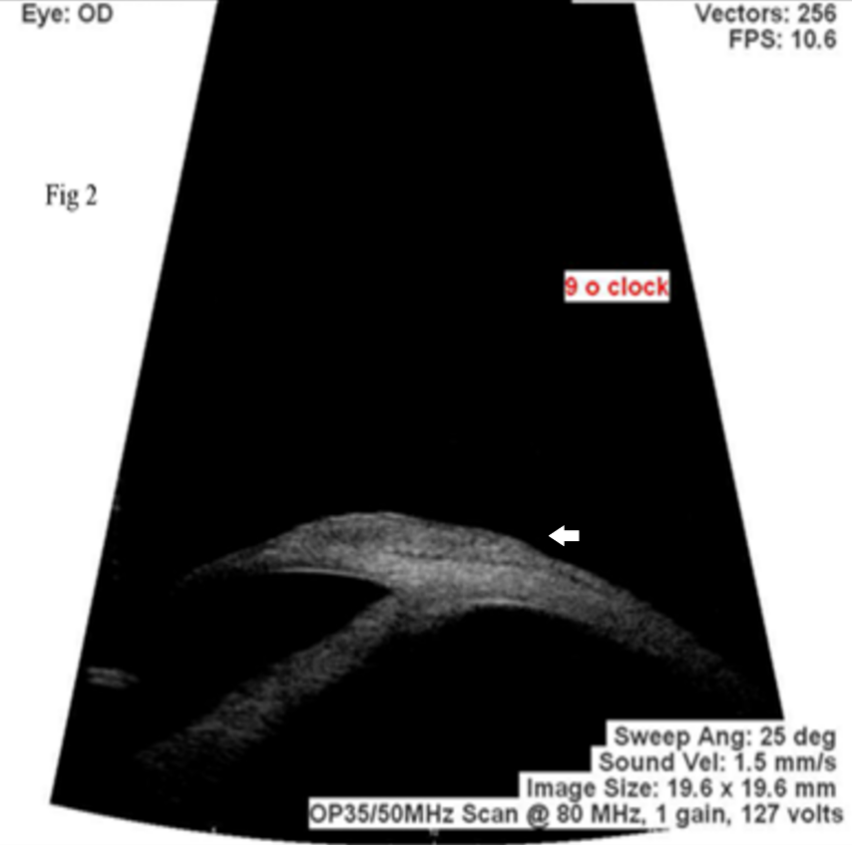
Ultrasound biomicroscopy (UBM) showing a hyperreflective mass (arrow) is seen from 7 o’clock to 2 o’clock in perilimbal area encroaching the cornea. The mass is in flush with conjunctiva and involving superficial and anterior stromal layers of cornea. Sclera and other uveal structures are not involved.

**Figure 3:**
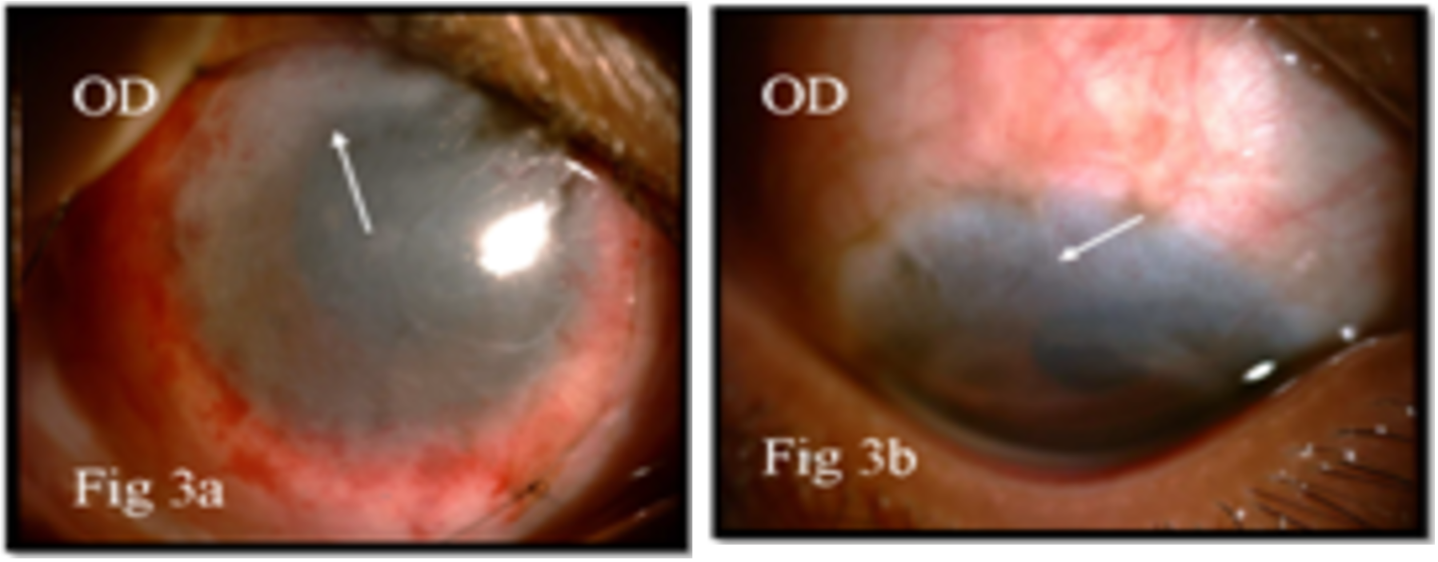
Right eye showing amniotic membrane (arrow) over excised surface post operatively, with purse string suture and bandage contact lens in situ (a). Right eye showing superior nebular corneal opacity on 6 months follow up (arrow) (b).

**Figure 4:**
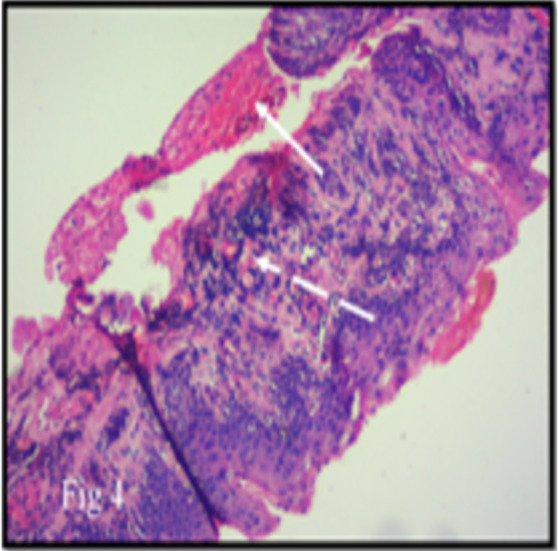
Haematoxylin and eosin stain showing acanthotic multilayered thickening (arrow) of the conjunctival epithelium with reduced goblet cells. The stroma shows fibrovascular tissue (interrupted arrow) with loss of polarity in the epithelial cells. There is no break in the basement membrane (magnification 40 X).

On examination, best corrected visual acuity was 6/9 in right eye and 6/6 in left eye. The right eye showed a gelatinous lesion extending from 7 to 2 o’clock limbus with vascular fronds on surface, with 3 mm corneal encroachment with feeder vessels and pseudogerontoxon (Figure 1a (Fig. 1)). Rose Bengal stain positivity was noted on the lesion (Figure 1b (Fig. 1)). No papilla or foreign body was noted on eversion of upper lid. Left eye showed pseudogerontoxon with no papillary and limbal activity (Figure 1c, d (Fig. 1)). The rest of the ophthalmic examination was unremarkable in both eyes. Right eye limbal lesion mimicking OSSN was suspected and impression cytology was done which showed multiple pigment bearing pleomorphic plump epithelial cells suggestive of OSSN. Two cycles of mitomycin C eye drop (0.04%) were given, which resulted in decreased size. Ultrasound biomicroscopy of the lesion showed no scleral involvement (Figure 2 (Fig. 2)). Anti-HIV 1 and 2 antibody test was negative. Excision biopsy of the lesion was done in toto with wide margin (4 mm) along with alcohol assisted removal of corneal lesion with no touch technique, dry surgical field with cryotherapy of margins and amniotic membrane transplantation (Figure 3a (Fig. 3)). The histopathology report revealed a multilayered thickening of the conjunctival epithelium with loss of polarity, nuclear atypia with no break in the basement membrane. The stroma showed fibrovascular proliferation suggestive of OSSN mild to moderate dysplasia (Figure 4 (Fig. 4)). At 6 months follow-up, visual acuity was 6/6 in both eyes and there was no evidence of recurrence of lesion (Figure 3b (Fig. 3)). 

## Discussion

PEH in VKC is a benign reaction and usually involves the superior limbus in the form of conjunctival thickening which often presents as gelatinous appearance, attributed to microtrauma caused by papillary reaction and inflammation [9]. Schwab et al. have previously reported the occurrence of a nodular limbal mass lesion in a case of limbal VKC which was associated with hypertrophic conjunctival thickening present superiorly [10]. When arising at the limbus, it may be difficult to clinically differentiate it from OSSN. One differentiating feature is the lack of capillary fronds in PEH while these may be seen in cases of squamous neoplasia or papilloma [8]. Malhotra et al. reported a rare limbal pseudoepitheliomatous hyperplasia mimicking OSSN in palpebral VKC [8].

In our case, limbal gelatinous lesion with corneal encroachment with feeder vessels and the staining with Rose Bengal raised the suspicion of OSSN. Impression cytology and histopathology were consistent with a diagnosis of OSSN. We report a rare presentation of OSSN masquerading as PEH in chronic VKC. Awareness of the similarity of the two is important for ophthalmologists in view of difference in approach and treatment. 

## Conclusion

Squamous lesions of the cornea and conjunctiva are uncommon but demand appropriate attention due to the potential for visual loss and systemic morbidity and mortality. Excellent results can be achieved by early detection, investigations, followed by meticulous surgery and regular follow-up of the patient. This case highlights the importance of strong clinical suspicion and detailed cytological and histopathological examination for early diagnosis and management of OSSN.

## Notes

### Competing interests

The authors declare that they have no competing interests.
